# Patent landscape of nanotechnology – based nucleic acid delivery systems for breast cancer therapies

**DOI:** 10.3389/fonc.2025.1702392

**Published:** 2026-01-14

**Authors:** Milca de Jesus Silva, Katharine Valéria Saraiva Hodel, Larissa dos Santos Moraes Fonseca, Helena Souza da Hora, Thiago Barros Murari, Claudio Damasceno Pinto, Bruna Aparecida Souza Machado

**Affiliations:** 1 SENAI CIMATEC University, SENAI Institute of Innovation (ISI) in Health Advanced Systems (CIMATEC ISI SAS), SENAI CIMATEC University, Salvador, Bahia, Brazil; 2 SENAI CIMATEC, Postgraduate Program in Industrial Management and Technology, SENAI CIMATEC University, Salvador, Bahia, Brazil; 3 FIOCRUZ, Oswaldo Cruz Institute (IOC), Rio de Janeiro, Brazil

**Keywords:** breast cancer, nucleic-acid therapies, patents, delivery-systems, therapies

## Abstract

**Background:**

Breast cancer is the most prevalent malignancy among women worldwide and remains a major public health concern. Conventional treatments are often limited by adverse effects and resistance. Nucleic acid-based gene therapies, supported by nanoparticle delivery systems, offer target and efficient therapeutic alternatives. The growing number of related patents about nanotechnology-based nucleic acid delivery systems for breast cancer treatment highlights the importance of understanding this landscape to advance research and innovation. Therefore, this study aimed to analyze the global patent landscape related to nanotechnology-based delivery systems for nucleic acid therapies in breast cancer, highlighting key technological platforms, emerging trends, and opportunities for research and development.

**Methodology:**

A patent landscape analysis was conducted using the Derwent World Patents Index (DWPI, Clarivate Analytics). The search strategy combined keywords and International Patent Classification (IPC) codes related to breast cancer, nucleic acid therapies, and delivery platforms. A total of 1,084 patent families were identified. Filters were applied to classify documents as alive, dead, or indeterminate, focusing on active patents from the past five years (2020–2025), resulting in 323 patents selected for details examination.

**Results:**

The analysis revealed a substantial increase in patent filings over the past decade, with the United States, China, and Europe leading technological innovations. Lipid-based nanoparticles and polymeric carriers were the most frequently cited platforms. The patents showed a strong focus on targeted delivery strategies, combinatorial approaches, and RNA interference technologies.

**Discussion:**

The findings highlight the transformative potential of nanotechnology for nucleic acid therapies in breast cancer. Despite manufacturing and regulatory challenges, the patent landscape demonstrates a dynamic and competitive field. Mapping technological developments provides valuable insights to guide strategic decisions in research and development and to identify underexplored areas with high innovation potential.

## 1 Introduction

**Figure 1:**
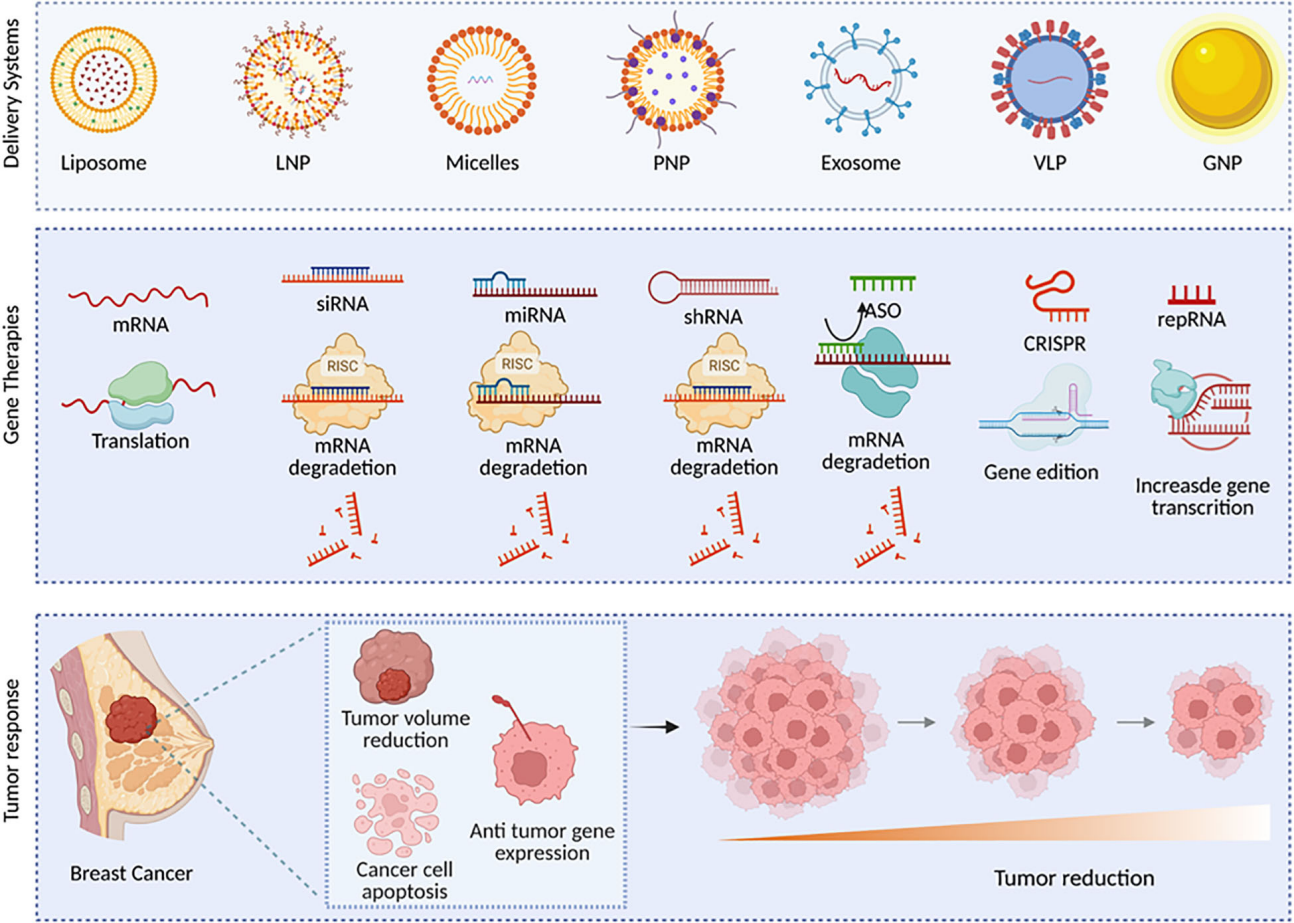
Delivery systems and gene therapies targeting breast cancer. Delivery systems, including Liposomes, Lipid Nanoparticles (LNP), Micelles, Polymeric Nanoparticles (PNP), Exosomes, Virus-Like Particles (VLP), And Gold Nanoparticles (GNP) enable efficient transport of nucleic acid-based therapeutics. The main therapeutic strategies using nucleic acids involve mRNA for translation; RNA interference such as siRNA, miRNA, and shRNA, acting through the RISC complex to degrade mRNA; antisense oligonucleotides (ASO); CRISPR-mediated gene editing; and self-amplifying RNA (saRNA), which boosts gene expression. Which leads to therapeutic outcomes in breast cancer, including tumor volume reduction, apoptosis of cancer cells, and expression of antitumor genes, leading to progressive tumor shrinkage. mRNA: messenger RNA; siRNA: small interfering RNA; miRNA, microRNA; shRNA, short hairpin RNA; ASO: antisense oligonucleotide; CRISPR: clustered regularly interspaced short palindromic repeats; saRNA: self-replicating RNA; RISC: RNA-induced silencing complex.

Breast cancer (BC) is the most prevalent cancer among women and represents a leading cause of cancer-related mortality, morbidity, and disability worldwide (1). As a result, BC remains a major global public health concern (2). According to the World Health Organization (WHO), approximately 2.3 million women were diagnosed with breast cancer in 2020, and the disease accounted for an estimated 685,000 deaths globally in the same year (3). The precise mechanisms underlying the initiation of breast cancer remain unclear (4, 5). However, it is well established that BC is a multifactorial disease influenced by both genetic predispositions and environmental exposures, which together contribute to the onset and progression of distinct clinical and molecular phenotypes (**Figure 1**) (4).

At the molecular level, BC encompasses a heterogeneous group of malignancies originating in the mammary glands (6), resulting in a classification based on a combination of molecular and histopathological features (5). Broadly, breast cancer is categorized into three main subtypes: tumors expressing hormone receptors—estrogen receptor (ER+) and/or progesterone receptor (PR+); tumors overexpressing the human epidermal growth factor receptor 2 (HER2+); and triple-negative breast cancer (TNBC), characterized by the absence of ER, PR, and HER2 expression (ER−, PR−, HER2−) (7, 8).

In summary, molecular classification, primarily based on gene expression and immunohistochemical profiling, has identified five major subtypes: luminal A, luminal B, HER2-enriched, basal-like, and triple-negative breast cancer (TNBC). Luminal A tumors are characterized by strong estrogen receptor (ER) and progesterone receptor (PR) expression, low proliferation index (Ki-67), and absence of HER2 amplification, typically associated with favorable outcomes and responsiveness to endocrine therapy. Luminal B cancers, in contrast, exhibit lower ER expression and higher proliferative activity, often with HER2 co-expression, leading to more aggressive phenotypes and the need for combined hormone and chemotherapeutic approaches (9). HER2-enriched tumors display overactivation of the HER2 signaling pathway, mediated by the amplification of the *ERBB2* gene, which promotes uncontrolled cell proliferation through downstream cascades such as PI3K/AKT and MAPK.

Basal-like and TNBC subtypes represent the most aggressive molecular classes, frequently lacking ER, PR, and HER2 expressions. Although TNBC and basal-like tumors share similar immunophenotypic profiles, they are not completely overlapping at the transcriptomic level; approximately 70–80% of TNBCs exhibit basal-like gene signatures. These tumors are enriched for mutations in *TP53*, *BRCA1*, and *PTEN*, leading to genomic instability, defective DNA repair via homologous recombination, and dysregulation of cell-cycle checkpoints. Consequently, TNBC progression is often associated with the activation of oncogenic pathways, including PI3K/AKT/mTOR, EGFR, and Wnt/β-catenin, which contribute to their invasive and metastatic potential. Due to the absence of actionable hormone or HER2 targets, chemotherapy remains the mainstay of systemic treatment for TNBC, although recurrence and metastasis rates remain high (10).

The molecular stratification of breast cancer therefore plays a pivotal role in guiding personalized treatment decisions. Immunohistochemical evaluation of ER, PR, HER2, and Ki-67 markers remains an essential diagnostic standard. However advances in genomic profiling have deepened the understanding of tumor heterogeneity and revealed novel molecular targets. This growing knowledge of breast cancer subtypes and signaling pathways underscores the need for innovative therapeutic modalities, including RNA-based nanomedicines capable of modulating key oncogenic pathways with precision and reduced systemic toxicity (11).

The molecular subtype of BC can influence various aspects, including the treatment approach, which also considers factors such as the tumor stage, histological grade, and the overall health status of the affected individual (12). Traditionally, BC treatment has focused on systemic therapies such as chemotherapy, hormonal therapy, targeted therapy, and immunotherapy (13). However, these treatment modalities are often associated with significant adverse effects and the development of drug resistance (14). Effective BC treatment must consider the heterogeneity of the disease, with emphasis placed on biologically driven therapies tailored to the molecular characteristics of the tumor, aiming to reduce the side effects associated with conventional treatments (5).

Accordingly, therapeutic strategies should therefore be guided by the specific molecular profile each tumor (15). In this context, genetic therapies have emerged as promising approaches for cancer management, given the strong genetic basis of tumor initiation and progression (16). Nucleic acid-based therapies (including DNA, microRNA, short hairpin RNA (shRNA), small interfering RNA (siRNA), self-amplifying RNA (saRNA), CRISPR/Cas systems, messenger RNA (mRNA), and antisense oligonucleotides (ASO)) offer the potential to correct mutated genes, suppress oncogene expression, and restore tumor suppressor pathways (17–23). Moreover, by targeting genes involved in cell-cycle regulation and checkpoint protein expression, these therapies have demonstrated the ability to modulate cell proliferation and induce apoptosis in breast cancer cells, particularly those associated with resistance to conventional treatments (14).

Despite their therapeutic potential, the clinical application of nucleic acids is limited by challenges related to their negative charge, susceptibility to enzymatic degradation and poor cellular uptake (24). To overcome these barriers, efficient and safe delivery systems are required to ensure targeted transport and intracellular release of these molecules (25). In this context, nanoparticles (NP)-based delivery has emerged as promising pharmaceutical carriers capable of enhancing delivery efficiency and specificity (24). Owing to their small size (typically <1000 nm) and modifiable surface properties, NPs can penetrate tumor tissues and selectively accumulate in cancer cells, reducing off-target effects and minimizing damage to healthy tissues and organs (4, 13, 24, 26, 27).

Furthermore, NP-based delivery strategies utilize various types of nanoparticles, such as liposomes, lipid nanoparticles (LNPs), micelles, polymeric nanoparticles, exosomes, metal-based particles, and virus-like particles, to transport therapeutic agents directly to tumor sites (24, 28–33). The significance of this technology has been acknowledged by the National Cancer Institute of USA, which recognizes nanotechnology as a paradigm-shifting advancement in the diagnosis and treatment of cancer (34).

As a result of these advancements, numerous patents involving nucleic acids and gene delivery systems have been developed in recent years for the treatment of breast cancer (35–37). The growing number of patent applications highlights the global effort to translate molecular innovations into clinically and commercially viable solutions.

In this context, intellectual property (IP) plays a key role in protecting and incentivizing the development of technologies related to gene therapy and delivery systems. The analysis of patent documents is a valuable tool that allows for the identification of technological trends, emerging therapeutic strategies, and market interest, thereby providing a strategic perspective on the translational potential of research in this field. Moreover, understanding the current patent landscape is essential for researchers, clinicians, and stakeholders aiming to advance novel therapies from the laboratory to clinical practice. This study provides an up-to-date understanding of technological trends and an in-depth analysis of Research and Development (R&D) scenarios. Such insights can support decision-makers in formulating innovation strategies, as well as identifying emerging or pre-commercial technologies, contributing to strategic positioning in a dynamic and competitive environment (38–41).

Therefore, the aim of this study is to provide an overview of patent documents involving nucleic acids and gene therapy delivery systems, offering a comprehensive perspective on current technological approaches and emerging trends in breast cancer gene therapy.

## 2 Materials and methods

A combined qualitative and quantitative approach was adopted to support the patent analysis related to gene therapy delivery systems in breast cancer. Patent data were retrieved from Derwent World Patents Index from Clarivate Analytics (covered by the license from SENAI CIMATEC University). The Derwent World Patents Index (DWPI) is an expertly curated patent database covering over 127 million documents from more than 60 jurisdictions (42). It is widely used by companies across a range of industries, as well as by research centers and patent examiners worldwide.

A search strategy was developed using advanced command lines to explore titles, abstracts, and indexed terms (Enhanced Patent Data - DWPI Only) and

Patent Collections by Authority, associated with delivery systems for gene therapies targeting breast cancer. The keywords used in formulating the search strategy were selected based on scientific literature relevant to the subject. The following Boolean query was used:

(“nanotechnology*” OR “nanoparticle*” OR “nanocarrier*” OR “drug delivery system*” OR “liposome*” OR “micelle*” OR “polymeric nanoparticle*” OR “nanoemulsion*” OR “liposome*” OR “exosome*”)

AND

(“breast cancer*” OR “breast neoplasm*” OR “BRCA2*” OR “BRCA” OR “mammary neoplasm*” OR “mammary tumor*”)

AND

(“gene therapy*” OR “gene silencing” OR “genetic therapy*” OR “antisense oligonucleotide*” OR “siRNA” OR “miRNA” OR “RNA interference” OR “CRISPR*” OR “CRISPR interference*” OR “genetic inhibition*” OR “small interfering RNA” OR “shRNA” OR “RNA*” OR “iRNA*” OR “microRNA*” OR “ASO*”)

Moreover, the International Patent Classification (IPC) provides a hierarchical system of language-independent symbols for the classification of patents and utility models according to the different areas of technology to which they pertain (42). Additionally, IPC codes related to drug delivery systems and gene therapies for breast cancer, as shown in **Table 1**, were included to refine the search strategies. For the patent search, IPC- (IPC Any) codes were also applied. Initially, only IPC codes were used; subsequently, these were combined with keyword-based search terms using the Boolean operator AND to improve precision and coverage. The following classification codes were selected based on their relevance to pharmaceutical compositions, biotechnology, and computational applications in healthcare:

**Table 1:** IPC codes used to refine the search strategy for patents related to drug delivery systems and gene therapies in breast cancer.

IPC Code	Description
A61P35/00	Antineoplastic agents
A61P35/02	Antineoplastic agents active against tumors of the blood
A61P35/04	Antineoplastic agents active against solid tumors
A61K9/00	Medicinal preparations characterized by special physical form
A61K9/127	Liposomes
A61K9/14	Microcapsules
A61K9/51	Nanocapsules
A61K47/48	Liposomes
A61K47/68	Micelles
A61K47/69	Nanoparticles

(A61P35/00 OR A61P35/02 OR A61P35/04 OR A61K9/00 OR A61K9/127 OR A61K9/14 OR A61K9/51 OR A61K47/48 OR A61K47/68 OR A61K47/69 OR A61K31/00 OR A61K39/00 OR C07K16/00).

Subsequently, the data were analyzed and interpreted through the identification of key indicators, including the publication year and projected expiration date, the leading countries responsible for the filings, as well as the principal applicants (companies, institutions and inventors involved). The asterisk symbol (*) was used to retrieve both singular and plural forms of a term, while quotation marks (“ “) were employed to identify exact phrases or fixed multi-word expressions (**Table 1**). The patent landscape analysis was conducted on March 28, 2025, retrieving a total of 1,084 DWPI families, corresponding to 1,084 individual patent records. After retrieving the initial set of documents, filters were applied to identify the most recent publications, remove duplicate patents, exclude patents lacking the specific search terms, and classify the remaining patents into three categories: alive, dead, and indeterminate. Moreover, the patent analysis was performed by reviewing the fields in Excel spreadsheets; however, no duplicated patents were identified or excluded, with the aim of assessing their relevance and categorizing the inventions in accordance to the research objectives of this study. Based on this analysis, the most relevant patents related to the application of nanotechnology in breast cancer therapy were selected, totaling 1,084 documents deemed pertinent to the scope of this work.

For the in-depth analysis, only active patents published in the last five years (2020–2025) were selected (323 patent documents), as this period reflects the most recent technological developments in the field. In contrast, all retrieved documents were considered for the overall trend analysis. Graphical representations were generated using GraphPad Prism version 13 for Windows (GraphPad Software, San Diego, California, U.S.A.), and visual figures were created using BioRender.

## 3 Results

### 3.1 Patent landscape: global distribution of patents and strategic R&D trends

Over the past few decades, there has been a gradual yet significant increase in the number of registered patents related to genetic therapies employing delivery systems for breast cancer treatment (43, 44). This trend underscore the growing global research efforts and technological advancements in this field, as evidenced by the increasing number of scientific publications and patent filings in recent years (35, 44, 45). Such expansion is primarily driven by the potential industrial applications of nucleic acid-based therapies and delivery technologies, as well as by the urgent need to address breast cancer as an escalating global health concern (22, 46, 47).

**Figure 2:**
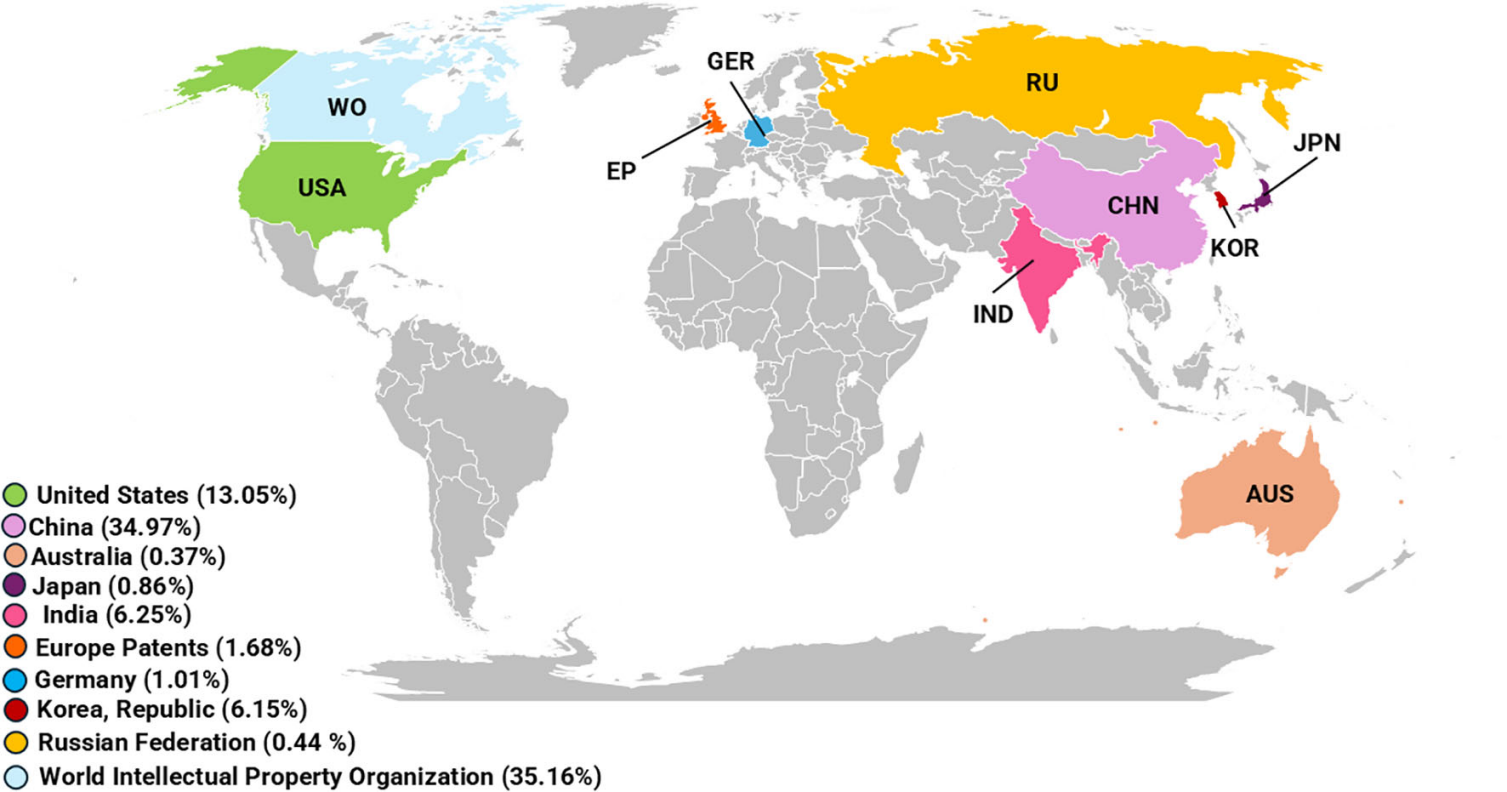
Global patent activity in genetic therapies using delivery systems for breast cancer. Top contributing countries/regions in patent publications. Leading patent assignees. EP: European Patent Office; WO: World Intellectual Property Organization.

The geographical distribution of patents reveals that China (368 patents – 34.97%), the United States (140 patents – 13.05%), and international patents filed under the World Intellectual Property Organization (WO) (370 patents – 35.16%) are the leading contributors in terms of the number of patent filings (**Figure 2A**). India follows with 67 patents (6.25%), further demonstrating the increasing global interest in genetic therapies for breast cancer across both developed and emerging economies (1, 4, 5, 48) (**Figure 2A**).

Furthermore, this concentration of patents within specific countries reflects not only strategic investments in innovation and health technologies but also correlates with the high incidence of breast cancer in these regions. Although breast cancer incidence is rising worldwide, industrialized nations continue to report the highest rates (5). Approximately half of all global breast cancer cases occur in developed countries, where the disease constitutes a major public health challege (37, 48). This observation suggest a direct correlation between the prevalence of disease and technological investment, since societal needs serve as guiding for innovation policies and industrial R&D priorities (49).

In fact, when social needs are clearly identified, they can guide both governmental funding strategies and private-sector inovation (50). The high incidence of breast cancer in these countries stimulated the development of new therapeutic technologies, promoting investment in R&D and leading to t patents registration as a form of intellectual protection. Public policies related to health can also focus on diseases with high incidence, resulting in increased financial support from governments and collaboration with private industries (51). Consequently, incentives for innovation are directly associated with patent growth, as patent protection provides exclusive commercial advantages (52).

In this context, the large number of patents registered in China can be associated with the rising incidence of breast cancer. Despite historically lower incidence rates, recent studies have shown a consistent upward trend. By 2022, breast cancer incidence in Chinese women was expected to exceed 100 per 100,000 individuals, with a total of approximately 2.5 million women aged 35 to 49 affected by the disease (53). This increase, coupled with China’s large population, has positioned the country as a central player in the development and protection of novel breast cancer therapy. Data indicate that China ranks first globally in terms of breast cancer burden, with incidence and mortality rates showing continued growth (17.6% and 15.6%, respectively), even though the overall national rates (36.1/105 for incidence and 8.8/105 for mortality) remain relatively low on a global scale (54, 55). Notably, since the early 2000s, China has expanded biotechnology investments, particularly focus on healthcare, strengthening its position as a global hub for innovation and technology transfer (56).

Similarly, in the United States, breast cancer remains the most frequently diagnosed cancer among women (57). In 2018 alone, the country reported 234,087 new cases, reinforcing its pivotal in BC research and therapeutic development (2, 48). Moreover, while breast cancer is also the most common cancer among urban Indian women (179,790 new cases reported in 2020), accounting for approximately 10% of all cancer diagnoses (58), India’s contribution to technological innovation in this area remains comparatively limited. This disparity reflects the complex relationship between disease burden and national capacity for scientific and technological response, influenced by differences in infrastructure, investment in R&D, and innovation policy. Hence, the global distribution of patents mirrors not only the epidemiological impact of breast cancer and healthcare innovation priorities but also highlights the importance of intellectual property protection as catalysis for technological advancement (59, 60). Nonetheless, the Indian government has adopted several strategies to mitigate this gap. For example, recent amendments to Indian patent law have encourage innovation, including the incorporation of Artificial Intelligence (AI) in patent development processes (61). These initiatives demonstrate the government’s efforts to create a more attractive scenario for applicants, potentially positioning India as a promising player in the development of new technologies in the coming years.

Despite China holding the highest number of patent publications, U.S.-based institutions represent the majority of assignees, accounting for 101 filings (45.70% of the total). These include the University of California (35 patents – 15.84%), Abraxis Bioscience LLC (27 – 12.22%), the University of Texas (22 – 9.95%), and Northwestern University (17 – 7.69%). Chinese institutions contributed 99 filings (44.80%), with a strong academic representation from the China Pharmaceutical University (24 – 10.86%), Sichuan University (21 – 9.50%), Fudan University (18 – 8.14%), Jinan University (18 – 8.14%), and Zhejiang University (18 – 8.14%). The Korea Institute of Science and Technology also made a notable contribution, with 21 applications (9.50%). India, however, does not appear among the top institutional assignees (**Figure 2B**).

Academic and research institutions thus play a decisive role in driving technological innovation and patent generatiom (52). The data reinforce the strong participation of institutions from the United States and China in the development and protection of technologies, with South Korea also contributing significantly. Additionally, robust patent protection systems are recognized as essential mechanism to stimulate innovation by safeguarding returns on R&D investments (62, 63). However, this capacity is closely tied to country’s economic strenght, since sustained R&D requires substantial funding (43, 62). The core argument is that robust legal protection of patent rights increases the likelihood that innovators can appropriate the returns from their research efforts (60). This impact is especially significant in patent-intensive industries, where strengthened IP frameworks tend to generate greater R&D activity (62).

It is also important to recognize that, while most countries have enacted patent laws, significant differences exist in the scope and enforcement of IP protections—such as the definition of patentable subject matter, duration of protection, and procedures for legal recourse. These variations play a critical role in shaping the innovation landscape (64, 65).

### 3.2 Temporal trends and expiration status of patents in nanotechnology-based nucleic acids delivery for breast cancer

**Figure 3:**
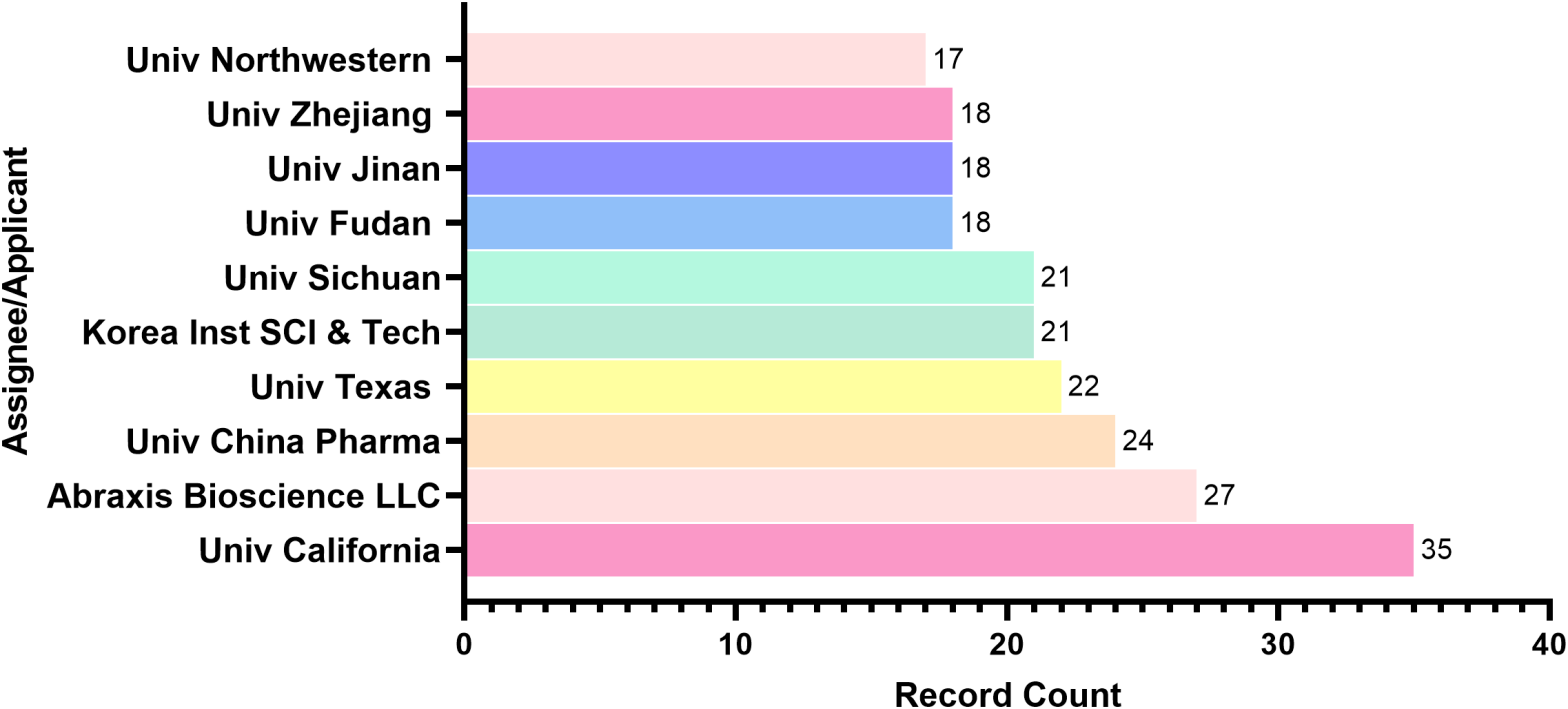
Patent Publication Trends and Expiration Timeline in Nanotechnology-Based nucleic acids Delivery for Breast Cancer annual publication trend of identified patents, illustrating the temporal evolution of technological development in the field Distribution of estimated expired patents, indicating the proportion of technologies whose legal protection period has ended.

The temporal distribution of patent activity indicated that between 2003 and 2015, the field experienced the lowest levels of patent activity, with a cumulative total of 723 patents, representing only 31.32% of the total publications from 2003 to 2025 (**Figure 3A**). This limited activity may be attributed to the dominance of traditional cancer therapies during that time, along with the emerging but not yet fully understood concepts of genetic therapies and nucleic acid delivery systems, particularly in the context of breast cancer (37, 66).

Indeed, a technological patents analysis conducted in 2015 demonstrated that the main types of treatment for breast cancer were surgery, radiation therapy, chemotherapy, and endocrine (hormone) therapy (37, 67).Additionally, although the concept of nucleic acids was already present, their use was primarily aimed at diagnosising phenotypic differences among breast cancers as a reflection of their mRNA expression profiles and defining molecular subtypes (67).

However, even during that period, studies had already emphasized the need for the development of selective drug delivery systems and innovative therapeutic carriers to overcome major limitations associated with conventional antineoplastic treatments, particularly systemic toxicity and poor bioavailability (39, 67–69). Although nanoparticles were indeed described as promising drug delivery systems, their application remained largely restricted to the drugs delivery systems (70).

Briefly, it is noteworthy that from 2017 to 2019, the number of patents appeared to plateau, totaling 498 filings over the three-year period, which corresponds to 21.56% of the total. This stabilization may suggest a phase of technological consolidation and optimization preceding a subsequent surge in innovation. In fact, between 2015 and 2019, all major universities in Europe and the United States experienced a decline in their positions within global scientific production rankings, which may partially explain the observed reduction in intellectual property filings during this period (71, 72) (**Figure 3B**).

In contrast, a significant increase in patent activity has been observed starting in 2020, with 207 publications, likely influenced by the global impact of the COVID-19 pandemic (45). The pandemic initiated a race to discover new treatments and preventive strategies for the disease, including the approval of the first mRNA vaccines that used lipid nanoparticles as delivery systems to target cells (73, 74). As a result of the successful application of nucleic acids using nanoparticle-based delivery systems, a new era of genetic therapies employing nanosystems has emerged, extending beyond infectious diseases to include non-infectious conditions as well (25, 75, 76).

Furthermore, it can be affirmed that the COVID-19 pandemic led to a major shift in how breast cancer services are managed and utilized (77). Since then, novels nanotechnology-based strategies have become essential for addressing complex therapeutic challenges in breast cancer treatment (31). Nanoparticle delivery systems have been increasingly proposed for nucleic acid transport and active delivery, with various therapeutic and technological objectives (78).

In this context, the success of nanoparticles as nucleic acid delivery systems is reflected in the number of patent publications in this field. Between 2020 and 2024, a total of 1,348 patents were published, accounting for 58.37% of all records—demonstrating a substantial surge in interest in genetic therapies based on nanoparticle delivery, particularly RNA-based approaches (17, 76, 79). This upward trend underscores the growing importance of nanotechnology in the delivery of nucleic acid therapeutics and the rapid advancement of gene-based strategies for cancer treatment (21, 53). In 2025, there have been 53 patent publications to March 28, 2025, representing 2.30% of the total, and this number is expected to increase as the year progresses (**Figure 3A**).

Additionally, an important aspect evaluated in this analysis was the estimated expiration year of the patents (**Figure 3B**). The data reveals that the highest number of patents already expired occurred in 2024, with 71 patents, representing 3.07% of the total, while 2032 had the lowest number of patent expirations, with 17 patents representing 0.74% (**Figure 3B**). In contrast, the greatest number of future expirations is projected for 2041, with 83 patents, corresponding to 3.59% of the total. After that, the number of expiring patents shows a gradual decline, reaching 30 patents in 2043, which accounts for 1.30% of all records.

The expiration of a patent signifies the end of exclusivity over a given technology, which then enters the public domain after the protection period (59, 60). This transition allows pharmaceutical companies to introduce generic versions of the original product, a process that plays a critical role in budget optimization by substantially reducing drug prices (43). Also, the introduction of generics or biosimilars after patent expiration contributes significantly to healthcare savings, especially within the first 1–5 years post-expiration (43).

The loss of exclusivity from originator drugs yields considerable benefits for patients, healthcare systems, and governments, as it enhances affordability and access to innovative therapies (80). For instance, South Korea has implemented a positive list system since 2007, ensuring that only cost-effective drugs are reimbursed, based on voluntarily submitted pricing and reimbursement applications by pharmaceutical companies (81).

Moreover, discussions about patent protection during public health emergencies—such as the COVID-19 pandemic—have resurfaced with intensity (82). The urgent need for population-wide immunization required rapid adjustments in public procurement systems to secure agreements with the pharmaceutical industry (83). During this time, the long-standing debate over the waiver of patent protections gained new relevance. On one side lies an ethical argument, advocating that the right to health and life supersedes intellectual property protection (82). On the other, there is the necessity to maintain mechanisms that stimulate innovation, as strong intellectual property rights (IPR) remain fundamental to the development and global positioning of national technological capacities (62, 82).

### 3.3 Therapeutic and/or technological aims in nucleic acid delivery for breast cancer

In addition to the temporal and geographical distribution of patent activity, it is essential to examine the specific therapeutic and technological objectives addressed by these innovations. For this purpose, the IPC codes serve as a valuable analytical tool, offering insight into the functional scope and intended applications of nanotechnology-based nucleic acid delivery systems for breast cancer treatment. These delivery systems are designed to protect nucleic acids from degradation during the delivery process. **Table 2** summarizes the main IPC codes identified in the analyzed patent documents and their respective descriptions.

**Table 2:** International Patent Classification (IPC) codes and their corresponding descriptions, as established by the World Intellectual Property Organization (WIPO), identified in prospective patent documents to indicate the technological focus.

IPC Code	Technological Focus	IPC (%)
A61P 35/00	Antineoplastic agents.	35.15%
A61K 9/127	Medicinal preparations in capsules containing microcapsules.	11.84%
A61K 9/51	Nanocapsules.	11.08%
A61K 9/14	Particulate form.	4.52%
A61K 47/69	Medicinal preparations containing polysaccharide-based carriers.	8.27%
A61K 41/00	Medicinal preparations are activated by wave energy or particle radiation.	7.52%
A61K 31/337	Medicinal preparations containing heterocyclic compounds with four-membered rings, e.g., paclitaxel.	6.17%
A61K 31/704	Medicinal preparations containing polysaccharides.	5.99%
A61K 47/34	Medicinal preparations containing lipid-based carriers.	5.27%
B82Y 5/00	Manipulation of nanoscale structures	4.26%

The analysis of IPC codes revealed that most patents related to gene therapy for breast cancer fall under A61P 35/00, which pertains to therapeutic compounds aimed at cancer treatment. This highlights a predominant focus on oncology within the patent landscape. Among delivery-related classifications, codes associated with nanoparticle-based systems were predominant, specifically, A61K 9/127 and A61K 9/51 refer to formulations involving liposomes and microspheres, respectively. The use of nanoparticles in anticancer drug delivery is supported by physiological mechanisms such as the enhanced permeability and retention (EPR) effect, which enables submicron particles to preferentially accumulate in tumor tissues (78).

Similarly, polymeric nanocarriers are represented by A61K 47/34, reflecting the use of polymer-based nanoparticles in breast cancer therapy. Accordingly, various hybrid polymeric systems have been developed to improve therapeutic efficacy (31). Additionally, regarding macromolecular carriers, A61K 47/69 represents macro-delivery platforms (typically larger than 1 mm), including implants, inserts, ocular patches, and matrices used in tissue engineering applications (84).

**Figure 4:**
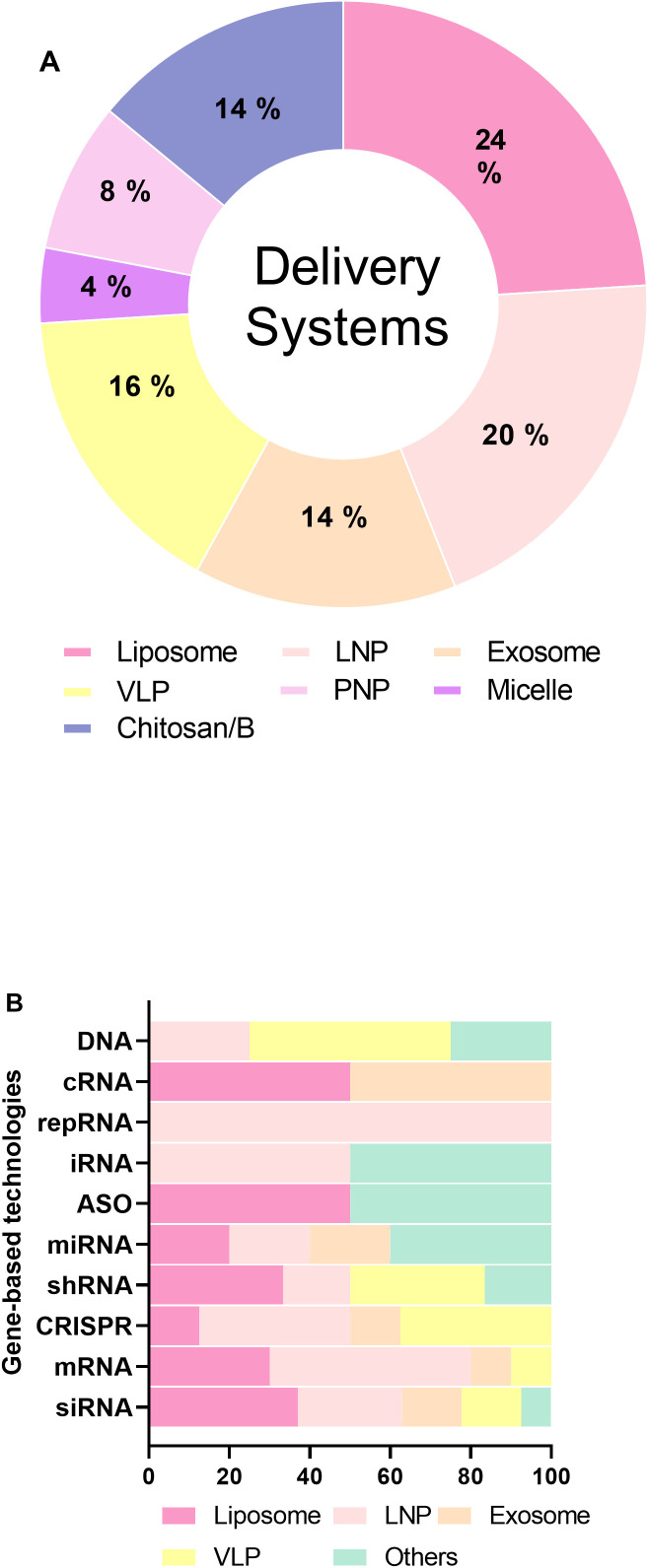
**(A)** Percentage distribution of gene-based therapies identified in the patent landscape. **(B)** Proportional representation of delivery systems found in the selected patents.

Moreover, controlled-release technologies also appear prominently in the dataset. The code A61K 9/14 corresponds to controlled-release dosage forms, indicating ongoing innovation in precision drug-release strategies. Likewise, A61K 41/00 encompasses pharmaceutical preparations characterized by special physical forms, further emphasizing advances in drug formulation and nanostructure design (**Figure 4**).

Additionally, the classifications A61K 31/337 and A61K 31/704 refer to specific chemical structures of active pharmaceutical ingredients, often associated with nucleic acids or modified sugars. These codes underscore the molecular specificity of many patented formulations, while B82Y 5/00 denotes the application of nanotechnology in medical or pharmaceutical contexts.

Collectively, these findings demonstrate a clear convergence among nanoscience and gene therapy, reflecting the deepening integration of molecular genetic into the development of next-generation therapeutic strategies (85, 86). The growing understanding of genetic material and its regulatory mechanism has significantly expanded therapeutic frontiers, particularly for diseases driven by inherited mutations or gene-regulated pathways (87).

Notably, the prevalence of nanocarrier-related IPC codes also underscores a translational movement toward multifunctional delivery systems, combining chemical stability, targeted delivery, and controlled release. These technological directions suggest that future RNA- and DNA-based therapies for breast cancer will increase rely on modular nanocarriers (e.g., LNPs, polymeric micelles, and hybrid lipopolyplexes) optimized for tumor selectivity and efficient intracellular trafficking, bridging the gap between patent innovation and clinical translation (88).

Beyond the descriptive mapping of IPC codes and patent counts, the current patent landscape reveals distinct technological breakthroughs that are reshaping the field of nucleic acid delivery for breast cancer. The prominence of nanocarrier-related classifications highlights a translational shift toward multifunctional systems capable of combining chemical stability, selective tumor targeting, and controlled RNA release (89). These advancements underscore the growing reliance on modular nanoplatforms—such as lipid nanoparticles (LNPs), polymeric micelles, and hybrid lipopolyplexes engineered for enhanced intracellular trafficking and reduced systemic toxicity (90).

Key industrial players, including BioNTech, Moderna, and Arbutus Biopharma, have established proprietary RNA delivery technologies that dominate both clinical pipelines and patent portfolios. Their platforms, particularly those based on ionizable lipids and optimized lipid compositions, have set the foundation for scalable, clinically validated RNA therapeutics. These industrial advancements reflect a global trend in which technological innovation and commercial investment converge to accelerate the translation of RNA nanomedicines from experimental to clinical stages (91–94).

Emerging therapeutic niches are also gaining momentum, especially those centered on bioinspired and extracellular vesicle (EV)-mediated RNA delivery. EVs represent a heterogeneous class of membrane-enclosed vesicles secreted by virtually all cell types, including cancer and immune cells. They carry diverse biological cargoes (e.g., RNAs, proteins, and lipids) that play key roles in intercellular communication, metabolic regulation, and immune modulation (95). The selective incorporation of RNA biotypes into EVs, including messenger RNAs, microRNAs, and circular RNAs, is now recognized as a tightly regulated process involving specific RNA-binding proteins and sorting machinery. This controlled loading enables EVs to act as natural carriers for functional RNA molecules, facilitating horizontal gene transfer and influencing the behavior of recipient cells (95, 96).

Recent studies have shown that non-coding RNAs (ncRNAs), particularly microRNAs and long non-coding RNAs, play a regulatory role in estrogen receptor (ER) signaling pathways that drive breast cancer progression. Nanotechnology-based delivery systems have been increasingly explored to modulate these ncRNA–ER interactions, enhancing treatment efficacy while minimizing systemic toxicity (90). Furthermore, exosome-based nanocarriers derived from breast cancer cells can achieve precise organotropic targeting and have been successfully functionalized to transport siRNA and microRNA molecules, enabling gene silencing in metastatic environments. Complementary advances in nanotechnology-based therapies, including liposomes, lipid nanoparticles, polymeric micelles, and hybrid nanocarriers, have further expanded the range of therapeutic tools for breast cancer. These systems not only improve drug solubility and tissue penetration but also allow active targeting and theragnostic applications, paving the way for personalized medicine. Artificial intelligence-driven design and bioinspired strategies are also being leveraged to optimize nanoparticle formulation and delivery performance (97).

Additionally, hybrid systems that integrate synthetic lipid layers with exosomal membranes further enhance delivery precision and biological performance, representing a new generation of biomimetic nanocarriers for breast cancer therapy. These technological and biological innovations demonstrate a paradigm shift from conventional synthetic nanocarriers toward adaptive, bioinspired delivery systems that better align with the complexity of tumor biology (88, 98, 99). As patent activity increases therapeutics, these approaches, RNA-based therapeutics are moving closer to achieving clinically meaningful outcomes in precision oncology.

### 3.4 Technological advancements and trends in nucleic acid delivery systems for breast cancer treatment

An additional layer of analysis focused on technological trends in patent innovations related to nucleic acid delivery systems for breast cancer reveals a notable predominance of RNA-based therapies over DNA-based approaches. This analysis was conducted using only active (alive) patents, as summarized in **Table 3**, which presents the most recent filings. A total of 49 active patents (~44.69%) were identified that involve the application of nanotechnology-based delivery systems for nucleic acids in breast cancer therapy. These include a range of nanoformulations designed to enhance cellular uptake, improve therapeutic index, and enable targeted delivery to tumor tissues. Full details of these patents are provided in **Supplementary Table 1**.

**Table 3:** Overview of gene therapy patents using nanotechnology-based delivery systems.

Publication No. / Date	Title	Delivery System	Gene Therapy	IPC Class	Country	Assignee	Ref
WO2025049877A1 / 03/06/2025	Chemo-Sensitive Dominant Clone For Adaptive Therapy	Virus-like Particle	mRNA	C12, A61, C07	WO	Moffitt Cancer Cent & Res Inst Inc H Lee	(82)
CN119345397A / 01/24/2025	RNA Delivery System For Directionally Programming Information Communication Between Cells	Exosome	CRISPR	A61, C07, C12	CN	Univ Sun Yat Sen Memorial Hospital Sun Y	(83)
CN119143643A / 12/17/2024	ROS response type ionizable lipid and use thereof for delivering nucleic acid	Lipid Nanoparticle	mRNA; DNA; siRNA; shRNA	C07, A61	CN	UNIV CHINA PHARM	(84)
WO2024219840A1 / 10/24/2024	Use of extracellular vesicles for targeted delivery of immune checkpoint inhibitor	Exosome	siRNA	A61, C12	WO	UNIV CATHOLIC KOREA IND ACADEMIC COOP	(85)
CN118767147A / 10/15/2024	Drug delivery system for treating malignant solid tumor based on attenuated salmonella	Virus-like Particle	ASO; siRNA; shRNA; miRNA	A61	CN	UNIV JILIN	(86)
WO2024205498A1 / 10/03/2024	Nanoparticle-based nucleic acid delivery system	Liposome	mRNA	A61, B01	WO	UNIV SINGAPORE NAT	(87)
WO2024182707A1 / 09/06/2024	Interleukin-2 and Interleukin-12 for cancer therapy	Viral and Non-Viral Carriers	DNA; mRNA; self-replicating RNA	A61, C07	WO	KRYSTAL BIOTECH INC	(88)
CN118531000A / 08/23/2024	Annular RNA and annular RNA medicine with anti-tumor function and application thereof	Lipid particles, sugar particles, metal particles, protein particles, liposomes, vesicles, exosomes, plasmid vectors, and viral vectors	circular RNA	C12, A61, C07	CN	UNIV NANJING CHINESE MEDICINE	(89)
WO2024128441A1 / 06/20/2024	Liposome-based drug carrier for immune anticancer therapy and method for producing same	Liposome	siRNA	A61, C12	WO	UNIV KONKUK IND COOP CORP	(90)
CN118121720A / 06/04/2024	Enzyme-responsive peptide-based nucleic acid targeted delivery system and preparation method	Micelle	siRNA	A61	CN	UNIV HEBEI TECHNOLOGY	(91)
CN117947024A / 04/30/2024	siRNA sequence for effectively inhibiting expression of complement C3 factor and application thereof	Lipid Nanoparticle	siRNA	C12, A61	CN	BEIJING JENKEM TECHNOLOGY CO LTD	(92)

CN, China; WO, World Intellectual Property Organization; US, United States; KR, South Korea; IN, India.

Furthermore, the patents analysis showed that iRNA-based technologies represent the dominant therapeutic modality among nucleic acid-based strategies, primarily aimed at gene silencing or editing, accounting for approximately 70% of the total. These approaches include the use of small interfering RNA (siRNA), microRNA (miRNA), antisense oligonucleotides (ASO), short hairpin RNA (shRNA), and CRISPR/Cas9 (**Table 4**). In this context, among siRNA-based technologies, self-amplifying RNA (saRNA) accounts for only around 6% of the patents related to RNA silencing, followed by ASO with 8%. The relatively low number of patents in these areas may indicate promising opportunities for innovation and technological investment. Conversely, the high number of patents involving iRNA suggests that this is perceived as a safer and more consolidated technology for investment. Such gene therapy techniques are considered among the most transformative advancements in modern medicine, as they are designed to edit human genetic material at the molecular level, repairing or replacing defective genes or silencing disease-associated targets (116, 117).

**Table 4:** Frequency of gene therapy technologies applied to breast cancer in the analyzed patent dataset.

Gene therapy	Technology description	Patents (%)	References
mRNA	Induces expression of therapeutic antitumor genes; inspired by COVID-19 success	20%	(21, 100)
CRISPR/Cas9	Precise genome-editing tool using Cas9 endonuclease and sgRNA	16%	(101–103)
shRNA	Triggers gene silencing via hairpin-loop RNA structures	12%	(22, 104)
miRNA	Endogenous regulators of gene expression via RISC complex; impact on epigenetic regulation	10%	(105)
ASO	Synthetic oligonucleotides that bind to mRNA or DNA to suppress gene expression	8%	(106–108)
siRNA	Synthetic double-stranded RNAs guiding RISC to degrade specific mRNA targets	11%	(23, 109–112)
saRNA	Self-replicating RNA that enhances intracellular protein expression	6%	(113, 114)
DNA and circular RNA (cRNA)	Modulate gene transcription	7%	(18, 115)

mRNA, messenger RNA; RNAi, RNA interference; siRNA, small interfering RNA; shRNA, short hairpin RNA; miRNA, micro RNA; CRISPR/Cas9, CRISPR-mediated gene editing; ASO, Antisense Oligonucleotide; saRNA, self-amplifying RNA.

In line with current clinical developments, these patent trends closely mirror the mechanisms of approved RNA therapeutics such as Onpattro^®^ (patisiran) and mRNA-1273, both employing LNP-based delivery systems for targeted gene silencing and protein expression, respectively (118, 119). Onpattro^®^, approved in 2018 by both the FDA and EMA as the first siRNA drug, demonstrated the clinical feasibility of siRNA–LNP systems by silencing transthyretin (TTR) mRNA in hereditary amyloidosis (120). Since then, multiple iRNA-based drug candidates have advanced to phase III clinical trials, highlighting the progression maturation of LNP-mediated delivery as a therapeutic platform (121).

Translating these delivery technologies into oncology, several siRNA and mRNA formulations have reached or are currently progressing through early-phase clinical evaluation (104, 122). For instance, neutral DOPC-based LNPs encapsulating EphA2-targeted siRNA (NCT01591356) are under phase I investigation for advanced solid tumors (123), while TKM-080301, targeting PLK1 (NCT02191878) (124), and CALAA-01 (125, 126) have demonstrated preliminary efficacy in solid and metastatic cancers (124–127). Similarly, preclinical studies in breast cancer models have shown encouraging outcomes using mRNA vaccines formulated in LNPs or saRNA systems targeting tumor-associated antigens such as HER2 and MUC1 (128, 129). In these models, LNP-encapsulated mRNA vaccines encoding MUC1 antigen induced potent cytotoxic T lymphocyte (CTL) responses and increased CD8^+^ tumor-infiltrating lymphocytes (TILs), particularly in triple-negative breast cancer (TNBC) models, thereby enhancing overall antitumor efficacy (130). Likewise, saRNA vaccines derived from alphaviral vectors expressing HER2 antigens triggered robust CD8^+^ T-cell activation and the production of HER2-specific antibodies, conferring durable immune protection and tumor regression in HER2^+^ murine models (131).

Notably, distinct molecular subtypes of breast cancer, such as HER2 overexpression, hormone receptor positivity, or the absence of these markers in TNBC, directly influence the choice of therapeutic RNA target and nanocarrier design (132, 133). For instance, HER2^+^ tumors may benefit from RNA formulations designed to elicit both antibody and T-cell responses against HER2 epitopes, whereas TNBC, characterized by high immunogenicity but limited targetable receptors, may be more responsive to mRNA vaccines encoding immunomodulatory or tumor-associated antigens such as MUC1 or IL-12 (21).

In this context, innovative RNA-based immunotherapy trials are exploring personalized and subtype specific strategies (134). One notable example is the Mutanome Engineered RNA Immuno-Therapy (MERIT, NCT02316457), which introduced the concept of Individualized Cancer Immunotherapy (IVAC^®^). This approach delivers patient-specific RNA vaccines designed to target neoantigens derived from mutanome of each tumor. In parallel, the WAREHOUSE model, developed by BioNTech, proposes a complementary strategy by maintaining an inventory of pre-manufactured RNA drug products targeting shared tumor-associated antigens (TAAs). Within this framework, the company has identified a set of immunogenic antigens recurrently expressed in TNBC, establishing a foundation for the development of standardized RNA-based therapeutics directed at this aggressive breast cancer subtype (135).

Furthermore, dendritic cell (DC)–based RNA vaccines have emerged as another promising platform. The NCT01291420 clinical trial investigates the immunogenicity and therapeutic potential of autologous RNA-modified DCs engineered to express the Wilms tumor 1 (WT1) antigen in patients with metastatic solid tumors, including breast cancer. Building upon earlier phase I results, this study hypothesizes that vaccination with WT1 mRNA-transfected DCs will be safe, well-tolerated, and capable of eliciting robust WT1-specific CD8^+^ T-cell responses, potentially translating into measurable clinical benefit (136).

Moreover, these experimental advances are complemented by growing patent activity focused on mRNA vaccine formulations for breast cancer, highlighting the translation of preclinical discovery into clinical application. For example, patent WO2021263081A2 describes a breast cancer vaccine encoding multiple tumor-associated antigens, including MUC1, HER2, hTERT, Survivin, MAGEA3, and Mammaglobin A, for use across molecular subtypes such as ER^+^, HER2^+^, and TNBC (132, 133, 137).

In this context, it is essential to emphasize that some of these patents represent not only academic or conceptual advances but also clinically applicable technologies already under translational development. The transition from intellectual property to therapeutic accessibility involves complex regulatory and economic processes, as successful patents entering clinical stages may lead to high production and commercialization costs. Therefore, ensuring that such innovations remain affordable and available to patients will require coordinated strategies for technology transfer, public–private partnerships, and pricing policies that balance innovation incentives with equitable access to treatment.

Additionally, mRNA vaccines encoding specific antigens or effector molecules can be rapidly and cost-effectively produced through *in vitro* transcription (IVT) from a DNA template. Recent technological progress in mRNA manufacturing and delivery, such as HPLC purification, incorporation of modified nucleosides, and complexation with carrier molecules, has markedly improved mRNA stability, translation efficiency, and safety (91, 138). Furthermore, a newer generation of saRNA vaccines has emerged, mimicking the structure of single-stranded RNA viruses, in which structural protein genes are replaced by antigen sequence while retaining the non-structural replicase genes (91). Once internalized into the cytoplasm, these replicons self-synthesize replicase enzyme, enabling *in situ* amplification of the RNA template and sustained antigen expression. This feature provides enhanced and prolonged immune stimulation compared to conventional mRNA vaccines, while maintaining a favorable safety profile due to the absence of infectious viral particles. Such platforms have demonstrated promising potential in cancer immunotherapy, offering long-lasting and reduced risk of adverse immune reactions (122).

Despite these promising developments, significant challenges persist in the delivery of nucleic acids for biomedical applications. A successful delivery system must protect therapeutic nucleic acids from degradation within the biological environment and ensure their targeted transport to specific tissues, cells, and subcellular compartments (139). In this context, the rational design and optimization of delivery vehicles remain critical for the advancement of gene therapy and RNA-based therapeutics (140). In this context, the emergence of lipid-based nanocarriers has revolutionized the systemic delivery of RNA molecules. For example, before the development of LNPs, siRNA molecules were typically administered as surface-bound lipoplex, relying on the enhanced permeability and retention effect for passive tumor accumulation (141). The encapsulation of siRNA into LNPs or PNPs significantly enhanced specificity and stability, circulation time, tumor uptake, gene silencing efficiency, thereby increasing therapeutic efficacy and minimizing off-target effects (142).

Also, the patents analysis demonstrates the predominant use of LNPs for mRNA (50%) and iRNA (50%) therapies, while liposomes are particularly favored for siRNA (37%), shRNA (33.3%), and ASO (50%) therapies. VLPs are most used for DNA (50%) and CRISPR (37.5%) therapies. Other systems, such as micelles and exosomes, are employed across a diverse range of therapies, with notable usage in miRNA (40%) and ASO (50%) delivery (**Figure 4B**). Briefly, viral and non-viral delivery systems have been employed in the administration of nucleic acid-based therapies (142, 143). Although viral vectors are renowned for their high transfection efficiency and stable gene expression, their clinical application remains limited due to safety concerns, such as immunogenicity and insertional mutagenesis (142). In this study, only 16% of the patents utilize viral vectors or virus-like particles (VLPs) for nucleic acid delivery, whereas 84% of the inventions favor non-viral nanocarriers as delivery platforms (**Figure 4B**).

Viral vectors continue to be widely studied for *in vivo* and *in vitro* gene therapy applications due to their efficient gene transfer (144). However, the advent and rapid development of nanotechnology has led to a shift in focus toward non-viral vectors, which offer enhanced safety profiles, modifiability, and targeting precision, positioning them as superior alternatives for gene delivery (145). Consequently, extensive efforts have been directed toward developing non-viral carriers with optimized biocompatibility, low toxicity, and effective delivery capabilities. These include liposomes, LNPs, and inorganic nanocarriers, which have demonstrated great promise in gene silencing therapies (142). Collectively accounts for 84% of the delivery systems described in the analyzed patents (**Figure 4A**), underlining their widespread application in breast cancer gene therapy.

Liposomes are vesicular structures formed by phospholipid bilayers capable of encapsulating both hydrophilic and hydrophobic drugs. They deliver genetic material by fusing cell membranes, a process that has been successfully exploited in various commercial transfection agents such as Lipofectamine^®^ and Oligofectamine^®^. Their aqueous core, enclosed within a phospholipid bilayer, is especially suited for encapsulating and protecting siRNA molecules from enzymatic degradation, facilitating tumor cell uptake, and promoting endosomal escape to ensure efficient cytoplasmic delivery (142, 146). Similarly, LNPs have gained significant attention due to their targeting capability, biocompatibility, and ability to reduce systemic toxicity, overcome drug resistance, and enable endosomal escape (147). LNPs are currently utilized in several commercial formulations, including LipofectamineTM, TurboFectTM, and StemfectTM, further validating their role in advanced gene therapy applications (87).

Furthermore, the patent analysis further illustrates specific applications of various nucleic acid delivery systems for breast cancer treatments. For instance, patents such as US20220127603A1, CN114015674A, and CN112410377A utilize liposomes for CRISPR delivery, emphasizing the potential of this technology in gene editing for breast cancer therapy. These liposomal delivery systems are highly favored due to their ability to encapsulate nucleic acids and facilitate efficient cellular uptake and endosomal escape. Similarly, US20190002889A1 demonstrates the use of liposomes for CRISPR-based therapies, showcasing their robust applications in gene editing for tumor suppression (**Table 3**). In addition, WO2024205498A1 focuses on the use of liposomes for mRNA delivery, highlighting their role in enhancing protein expression in targeted cells. WO2024128441A1 describes liposomal systems designed for the effective delivery of siRNA, contributing to gene silencing in cancer cells. Additionally, CN117402873A explores liposomes for ASO therapies, which work by binding to target mRNA and inhibiting gene expression at the transcriptional level.

For LNPs, WO2024078645A2 describes their use in mRNA or CRISPR delivery, demonstrating their biocompatibility and ability to facilitate gene editing and protein production. Likewise, WO2024078534A1 utilizes LNPs for siRNA delivery, showcasing their efficiency in gene silencing. CN117286143A focuses on iRNA delivery via LNPs, while CN116617415A and WO2023097317A1 emphasize mRNA and saRNA delivery, respectively. Notably, other nucleic acid delivery systems represent 56% of the delivery technologies in the patents analyzed (**Figure 4A**). Exosomes, such as CN119345397A for CRISPR delivery, and WO2024219840A1 for siRNA delivery, have emerged as promising candidates for nucleic acid transport due to their natural ability to encapsulate and protect genetic material. Additionally, micelles, described in patents like CN118121720A and CN114224838A, are used for siRNA delivery, taking advantage of their amphiphilic nature for effective nucleic acid encapsulation.

VLPs have also been utilized for gene delivery in patents such as WO2023091696A1 for DNA delivery and WO2025049877A1 for mRNA delivery, both of which highlight their ability to efficiently deliver nucleic acids while mimicking viral behavior to enhance cellular uptake and transfection efficiency. Other delivery systems, such as metal nanoparticles, microvesicles, gene-gun technology, and polymeric nanoparticles, have also been explored in patents related to genetic therapies in breast cancer, with brief descriptions provided in **Table 2**. Overall, this analysis indicates a significant interest in the development of nucleic acid-based technologies, particularly those targeting gene silencing. The liposome and LNP carriers have emerged as promising vehicles for efficient nucleic acid delivery, further supported by the diverse technological advancements presented in the patents analyzed. This diversity of patent technologies highlights the great potential for the development of innovative therapies for breast cancer, utilizing advanced nanoparticle-based delivery systems.

## 5 Discussion

In conclusion, delivery systems in gene therapy have demonstrated remarkable potential in addressing breast cancer treatment, acting mainly on genetic mutations associated with the disease. This review highlights a growing and strategic interest in nucleic acid-based therapies, particularly those employing gene silencing mechanisms (siRNA, miRNA, shRNA, ASO) and gene-editing tools such as CRISPR/Cas9. Notably, 70% of the identified patents focus on silencing oncogenic pathways, reflecting a clear trend in therapeutic strategies and the pharmaceutical industry’s interest in developing patents in this area.

Moreover, the temporal analysis demonstrates that technological advancements in this field have accelerated significantly since 2020, largely influenced by the success of mRNA and nanoparticle-based delivery platforms during the COVID-19 pandemic, as a response to previous challenges related to bioavailability, toxicity, and delivery precision. These findings underscore the dynamic innovation landscape and support the potential of nanotechnology-driven gene delivery systems as transformative tools for future breast cancer treatment strategies. The predominance of non-viral delivery platforms in the patents analyzed may indicate the shift toward more adaptable and scalable solutions for gene therapy applications.

The increasing burden of non-communicable chronic diseases, particularly cancer, has driven the search for new therapeutic alternatives. Breast cancer, projected to see a 77% rise in new cases by 2050 (148), stands out as a major public health concern. In this context, RNA-based therapies have shown significant potential, especially following the advancements made during the COVID-19 pandemic, when mRNA technology proved effective against infectious diseases. Now, this technology is being repurposed for cancer treatment, offering the promise of more personalized and less invasive therapies. However, the clinical application of this technology faces challenges, such as the need for efficient delivery systems that protect RNA from degradation by naturally occurring enzymes, which limits its efficacy (149).

Nanotechnology, with nanostructured delivery systems, emerges as a promising solution to these challenges. Countries like China and the United States, which already prioritize breast cancer as a public health issue, have heavily invested in cutting-edge technologies in this field, accelerating the development and implementation of these therapies. Despite these advancements, critical areas remain to be improved, such as the stability of RNA therapeutics and the scalability of delivery systems. Ongoing research is essential to address these limitations, with a focus on optimizing nanoparticles and liposomes, which are already well-established in drug delivery (150).

Despite significant progress, several translation barriers still hinder their clinical implementation (88, 151). One of the most critical issues relates to the inefficient delivery of RNA molecules within the tumor microenvironment. Due to their negative charge and high molecular weight, RNA strands cannot readily cross the cellular membrane and are prone to enzymatic degradation by serum RNases and endonucleases. Nanocarriers have been widely explored to enhance stability and targeting efficiency. Nevertheless, endosomal escape, off-target effects, and non-specific accumulation in healthy organs (e.g., liver, spleen, and kidneys) remain major limitations that affect both therapeutic efficacy and biosafety (151).

Another critical translational bottleneck concerns the heterogeneity of the tumor microenvironment in breast cancer. Variations in vascularization, extracellular matrix density, and immune cell composition influence nanoparticle penetration and retention. This is particularly relevant in resistant subtypes such as HER2-positive and triple-negative breast cancer (TNBC), where dysregulated signaling pathways (e.g., PI3K/AKT/mTOR, HER2, and BRCA1/2 repair networks) drive aggressive phenotypes and drug resistance. Recent studies have demonstrated that circular RNAs (circRNAs) contribute to trastuzumab resistance by sustaining HER2 downstream signaling and impairing ferroptosis (105, 152). These findings highlight how molecular and immune mechanisms within the TME may compromise the efficacy of RNA-based nanotherapies, underscoring the need for combinatorial strategies that integrate immune modulation and targeted delivery (152, 153).

Furthermore, the immunogenicity and long-term safety of RNA-loaded nanocarriers must be carefully assessed (91, 92). While chemical modifications and encapsulation strategies mitigate innate immune activation, prolonged exposure or repeated dosing could still elicit undesired inflammatory responses. Strategies that integrate immune-evasive materials and surface functionalization with targeting ligands (e.g., peptides or antibodies) may enhance selectivity while minimizing toxicity. Moreover, ensuring endosomal escape and cytoplasmic release without compromising cellular integrity remains a key engineering challenge for nanocarriers (88, 154).

While biological complexity limits therapeutic efficacy, scaling up nanocarrier production under Good Manufacturing Practice (GMP) conditions poses additional technological challenges (155). The physicochemical properties of nanomaterials must be tightly controlled to ensure reproducibility, stability, and safety (155). Even minor deviations during synthesis can alter critical quality attributes, impacting the pharmacokinetics and biodistribution of the final product. The implementation of Quality by Design (QbD) frameworks has emerged as a promising approach to address these issues. QbD emphasizes the identification of critical process parameters (CPPs) and material attributes (CMAs) that influence the quality target product profile (QTPP), allowing continuous optimization during scale-up. However, regulatory guidance remains limited, with most existing FDA and EMA recommendations restricted to liposomal formulations (155). Broader regulatory harmonization is required to accelerate clinical translation and approval of advanced nanocarrier platforms, including lipid-based and bioinspired RNA delivery systems.

## 6 Future perspectives and challenges

Although significant progress has been made in RNA-based nanodelivery technologies, the production processes remain complex, time-consuming, and costly. Large-scale manufacturing under Good Manufacturing Practice (GMP) conditions still faces challenges in ensuring reproducibility, maintaining RNA stability, and achieving consistent encapsulation efficiency. These limitations hinder the rapid translation of promising nanocarrier formulations from the laboratory to clinical application in breast cancer therapy.

Computational modeling, artificial intelligence (AI), and machine learning (ML) have emerged as transformative tools to address these barriers. By simulating nanoparticle behavior, predicting RNA–nanocarrier interactions, and optimizing formulation parameters, these technologies can significantly reduce experimental costs and development timelines. Furthermore, AI-driven analysis of patent and omics datasets can uncover novel design patterns, guide rational formulation strategies, and identify innovation hotspots within the field of RNA therapeutics.

In parallel, the integration of bioinspired nanocarriers, such as engineered exosomes and cell membrane-coated vesicles, offers new opportunities to enhance tumor targeting, improve immune compatibility, and minimize off-target effects. The combination of mRNA vaccines, RNA interference, and CRISPR-based editing within advanced delivery systems holds great promise for overcoming therapeutic resistance and advancing personalized breast cancer treatment.

Looking forward, harmonized regulatory frameworks, scalable GMP manufacturing, and comprehensive toxicological profiling will be essential to ensure clinical translation. By coupling experimental nanotechnology with computational intelligence, RNA-based nanomedicines may soon achieve greater efficiency, lower production costs, and broader accessibility, paving the way for the next generation of precision oncology.

## Data Availability

The original contributions presented in the study are included in the article/**Supplementary Material**. Further inquiries can be directed to the corresponding author.
