# Supplementary material for: Patent landscape of nanotechnology – based nucleic acid delivery systems for breast cancer therapies
Source: Front Oncol. 2026 Jan 14;15:1702392. doi: 10.3389/fonc.2025.1702392 (PMC12847028; doi:10.3389/fonc.2025.1702392)
Supplement: Supplementary file 1 [file Table1.docx]

| **Table I.** Overview of Gene Therapy Patents Using Nanotechnology-Based Delivery Systems | | | | | | |  |
| --- | --- | --- | --- | --- | --- | --- | --- |
| **Publication No. / Date** | **Title** | **Delivery System** | **Gene Therapy** | **IPC Class** | **Country** | **Assignee** | **Ref** |
| WO2024078645A2 / 18/04/2024 | Cas protein and use thereof | Lipid Nanoparticle | mRNA; CRISPR | C12 | WO | GUANGZHOU REFORGENE MEDICINE CO LTD; ZHEJIANG SYNSORBIO TECH | [1] |
| WO2024078534A1 / 18/04/2024 | Interfering RNA for inhibiting expression of melanophilin (MLPH) gene, and application thereof | Lipid Nanoparticle | siRNA | C07, A61 | WO | BEIJING JENKEM TECHNOLOGY CO LTD | [2] |
| WO2024031000A2 / 08/02/2024 | Secreted RNA therapeutics for nuclear delivery | Virus-like Particle | mRNA | C12, A61 | WO | MOFFITT CANCER CENT & RES INST INC H LEE | [3] |
| CN117402873A / 16/01/2024 | Antisense oligonucleotide for inhibiting endonuclease expression and application thereof | Liposome | ASO | C12, A61 | CN | UNIV SICHUAN WEST CHINA SECOND HOSPITAL; CHENGDU JILUOKELIN | [4] |
| CN117286143A / 26/12/2023 | Interference RNA for inhibiting TUBB2 gene expression and application thereof | Lipid Nanoparticle | interfering RNA | C12, A61 | CN | BEIJING JENKEM TECHNOLOGY CO LTD | [5] |
| CN117257970A / 22/12/2023 | Delivery system for targeted inhibition of epidermal growth factor receptor | Viral Vector | siRNA | A61, C12 | CN | UNIV NANJING | [6] |
| CN116855497A / 10/10/2023 | siRNA and liposome for inhibiting human USP46 gene expression and application thereof | Liposome | siRNA | C12, A61 | CN | UNIV JINAN GUANGDONG | [7] |
| CN116656619A / 29/08/2023 | Engineering exosome for pancreatic cancer treatment | Exosome | siRNA | C12 | CN | UNIV SUZHOU FIRST AFFILIATED HOSPITAL | [8] |
| CN116656618A / 29/08/2023 | An exosome of PARP1/2 siRNA and application thereof in treating breast cancer | Exosome | siRNA | C12 | CN | UNIV SUZHOU FIRST AFFILIATED HOSPITAL | [9] |
| CN116650653A / 29/08/2023 | Method of down-regulating agent composition for direct reprogramming of tumour cells | Viral and Non-Viral Carriers | siRNA; shRNA | A61, C12 | CN | HAIMEN YULIN CELL TECHNOLOGY CO LTD | [10] |
| CN116617415A / 22/08/2023 | A lipid nanoparticle drug delivery system and preparation and application thereof | Lipid Nanoparticle | mRNA | A61 | CN | SHANDONG YIFAN MEDICAL TECHNOLOGY CO LTD | [11] |
| WO2023097317A1 / 01/06/2023 | Methods of generating self-replicating RNA molecules | Lipid Nanoparticle | self-replicating RNA | C07, C12 | WO | REPLICATE BIOSCI INC | [12] |
| WO2023091696A1 / 25/05/2023 | Adenovirus delivery system for cancer treatment | Virus-like Particle | gRNA; DNA; CRISPR | A61, C12 | WO | CHRISTIANA CARE GENE EDITING INST LLC | [13] |
| CN115920068A / 07/04/2023 | A nano drug delivery system of carrier band si-RNA for anti-trastuzumab-resistant breast cancer | Liposome | siRNA | A61 | CN | HANGZHOU BOYI BIOMEDICAL TECHNOLOGY CO | [14] |
| CN115778918A / 14/03/2023 | A gene nano-delivery system based on calcium-binding polysaccharide and calcium phosphate | Calcium-binding Nanoparticle | DNA; RNA; hybrid | A61, C12 | CN | UNIV CHINA PHARM | [15] |
| CN115702882A / 17/02/2023 | Cation liposome modified by cRGD and nucleic acid complex for anti-tumour | Liposome | shRNA | A61 | CN | UNIV JINAN GUANGDONG | [16] |
| US11510975B1 / 29/11/2022 | Compositions and methods for inducing ESR1, PI3K, HER2, and HER3 immune responses | Liposome; VLP; LNP; Polymer NP | self-replicating RNA | A61, C07, B82 | US | REPLICATE BIOSCI INC | [17] |
| CN114921439A / 19/08/2022 | CRISPR-Cas effector protein, gene editing system and application thereof | Nanoparticles; Liposome; Exosome | CRISPR | C12, A61, C07 | CN | YAOTANG SHANGHAI BIOTECHNOLOGY CO LTD | [18] |
| CN114681428A / 01/07/2022 | Delivery system for targeted inhibition of EGFR and application thereof | Viral Vector | siRNA | A61, C12 | CN | UNIV NANJING ARTIFICIAL INTELLIGENCE | [19] |
| US20220127603A1 / 28/04/2022 | Novel CRISPR RNA targeting enzymes and systems and uses thereof | Nanoparticle; Liposome; Exosome; Microvesicle; Gene Gun | CRISPR | C12, C07 | US | ARBOR BIOTECHNOLOGIES INC | [20] |
| CN114224838A / 25/03/2022 | Bionic nanometre delivery system activated by tumour microenvironment | Micelle | siRNA | A61, B82 | CN | SHANGHAI PULMONARY HOSPITAL | [21] |
| CN114015674A / 08/02/2022 | Novel CRISPR-Cas12i system | Nanoparticles; Liposome; Exosome | CRISPR | C12, A61 | CN | HUIDAGENE THERAPEUTICS (various affiliates) | [22] |
| CN113940920A / 18/01/2022 | Co-encapsulated siRNA and hydrophobic drug nano system | Hydroxyethyl starch polymer + siRNA | siRNA | A61, C08 | CN | HUST TONGJI MEDICAL COLLEGE UNION HOSPITAL | [23] |
| CN112941072A / 11/06/2021 | Nucleic acid self-assembly structure and preparation method | Polymers; Liposome; Inorganic NP | mRNA | C12, A61 | CN | NAT CENT NANOSCIENCE & TECHNOLOGY CHINA | [24] |
| KR2224580B1 / 08/03/2021 | Nucleic acid construct for simultaneous gene expression and suppression | Nanoparticle | siRNA; mRNA | C12, A61 | KR | UNIV EWHA IND COLLABORATION FOUND | [25] |
| CN112410377A / 26/02/2021 | VI-E and VI-F type CRISPR-Cas systems and use | Nanoparticle; Liposome; Exosome | CRISPR | C12, A61, C07 | CN | CHINESE ACAD SCI; HUIDAGENE & Affiliates | [26] |
| CN111265669A / 12/06/2020 | Nucleic acid delivery carrier for small interfering RNA | Polymer Nanoparticle | siRNA | A61 | CN | UNIV SOUTH CHINA TECHNOLOGY | [27] |
| KR2020026757A / 11/03/2020 | Drug and gene co-delivery system based on cell-penetrating adhesive protein nanoparticles | Iron Nanoparticle | interfering RNA | C07, A61 | KR | POSTECH ACAD-IND FOUND | [28] |
| CN110038025A / 23/07/2019 | RNA triple helix hydrogel for targeted therapy of triple negative breast cancer | Nanoparticle | miRNA | A61, C12 | CN | UNIV LINYI | [29] |
| CN109985249A / 09/07/2019 | Tumour-targeting gene delivery system of ROS-sensitive material | Cationic Polymer Delivery System | siRNA | A61 | CN | UNIV FUDAN | [30] |
| CN109503411A / 22/03/2019 | Tertiary amine cationic lipid derivative for RNA delivery | Liposome | siRNA | C07, A61 | CN | UNIV CHINA PHARM |  |
| KR2019030452A / 22/03/2019 | Bio-nanoparticle for inhibiting immune checkpoint | Biopolymer Nanoparticles | siRNA; shRNA; miRNA; ASO | A61, A23 | KR | UNIV KONKUK GLOCAL IND ACADEMIC COLLABOR | [31] |
| IN201931006560A / 15/03/2019 | Silica nanoparticles for delivery of miRNAs in TNBC | Silica Nanoparticles | miRNA | A61 | IN | ADHIKARY A |  |
| US20190002889A1 / 03/01/2019 | Novel CRISPR RNA targeting enzymes and systems | Nanoparticle; Liposome; Exosome | CRISPR | C12, C07 | US | ARBOR BIOTECHNOLOGIES INC | [32] |
| KR2018122128A / 12/11/2018 | Quantum dot-nucleic acid-aptamer-liposome complex | Liposome | siRNA; shRNA; miRNA; ASO | A61 | KR | UNIV YONSEI WONJU IND-ACAD COOP FOUND | [33] |
| CN107184987A / 22/09/2017 | Polypeptide nanoparticle carrier with integrin targeting | Polypeptide Nanoparticle | siRNA | A61 | CN | SHANGHAI CHANGHAI HOSPITAL | [34] |
| KR2017044324A / 25/04/2017 | siRNA gene delivery using chitosan-hyaluronic acid nanoparticles | Chitosan Nanoparticle with Hyaluronic Acid | siRNA | A61, C08, C12 | KR | UNIV KONKUK GLOCAL IND ACADEMIC COLLABOR | [35] |

CN: China; WO: World Intellectual Property Organization; US: United States; KR: South Korea; IN:India.
